# Chronic Distal Biceps Tendon Rupture With Allograft Reconstruction

**DOI:** 10.7759/cureus.30805

**Published:** 2022-10-28

**Authors:** Moaath A Alamir, Kholoud M Alotaibi

**Affiliations:** 1 Department of Surgery, College of Medicine, Imam Mohammad Ibn Saud Islamic University, Riyadh, SAU; 2 Department of Medicine, Princess Nourah Bint Abdulrahman University, Riyadh, SAU

**Keywords:** allograft, growth hormone, steroids, athletic injury, biceps tendon

## Abstract

Rupture of the distal biceps tendon typically occurs after an eccentric extension load is applied to the elbow. Chronic distal biceps ruptures are uncommon and are complicated by tendon, muscle retraction, and tissue atrophy. Here, we present the case of a 26-year-old male soldier. The patient was a smoker on steroids and growth hormones. He had a distal biceps tendon rupture for two months following weightlifting. He had a positive hook test, and the tendon could not be palpated in his antecubital fossa. Despite trying conservative treatment earlier, he complained of weakness and was unhappy with the cosmetic appearance of his arm. He underwent a successful distal biceps tendon reconstruction with an allograft. At the three-month follow-up after the surgery, the patient reported a full range of motion and strength and was able to return to his daily life activities. In addition, the aesthetic appearance of the biceps muscle was restored.

## Introduction

The distal biceps tendon is a paratenon-lined, extra-synovial structure [1]. The biceps brachii muscle flexes the elbow and supinates the forearm. There are two heads, namely, a long head that originates from the supraglenoid tubercle, and a short head that originates from the coracoid process, with a distal insertion on the radial tuberosity. An injury to the biceps is typically caused by eccentric contraction or resisted elbow flexion associated with heavy lifting or a fall onto outstretched hands. Complete rupture of the distal biceps occurs rarely, with an annual incidence of 1.2 per 100,000 people [2]. Moreover, approximately 86% of distal bicep ruptures in men between the ages of 30 and 50 years occur in the dominant arm [3], in association with a history of tobacco product or anabolic steroid use, which contributes significantly to the risk [2]. Most often, the deformity can be seen, and the biceps tendon cannot be felt in the antecubital space. Because of forceful elbow extension while the biceps are contracting, the patient experiences a popping or tearing sensation in the antecubital region which is the classical presentation. Here, we present the case of a chronic distal biceps tendon rupture in an adult smoker with a history of chronic use of systemic corticosteroids and growth hormones for bodybuilding goals.

## Case presentation

A 26-year-old male, soldier, smoker, known case of asthma on steroids as treatment, started bodybuilding and taking growth hormones and steroids. While lifting dumbbells, he felt a pop sound in his left elbow. He noted immediate bruising in his antecubital fossa with loss of supination and flexion strength. He did not report pain or swelling. At a nearby hospital, an X-ray and ultrasound were obtained which did not reveal a tendon tear. After one week, he underwent magnetic resonance imaging (MRI) that confirmed the diagnosis of distal biceps tendon rupture. Six weeks after the initial injury, he attended our clinic with a complaint of weakness.

**Figure 1:**
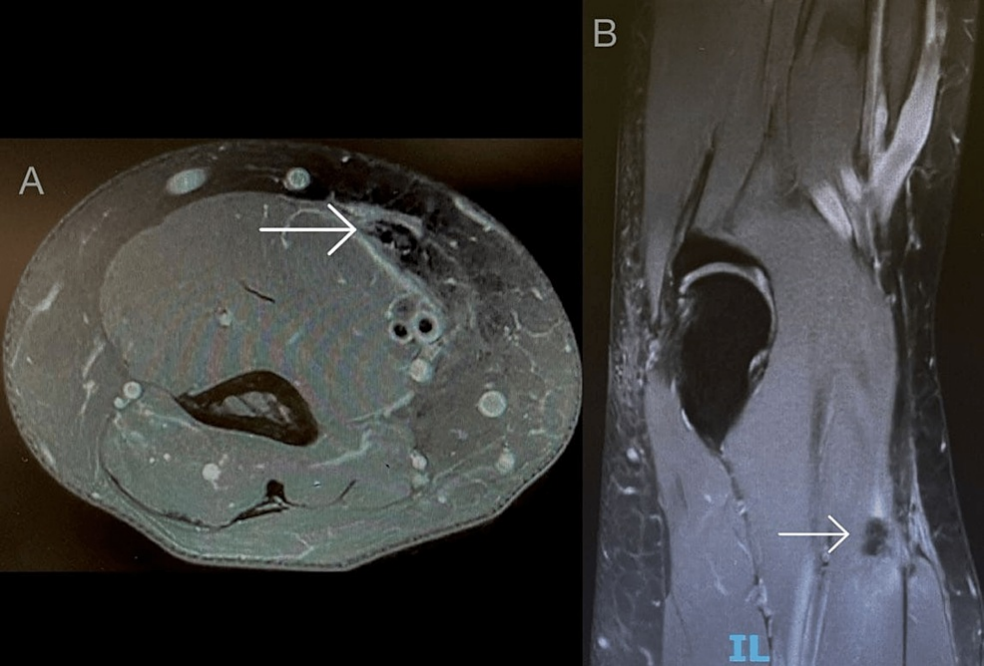
Magnetic resonance imaging findings. (A) Axial T2-weighted image showing the absence of the distal biceps tendon (arrow). (B) Sagittal T2-weighted image showing the retraction of the torn distal biceps tendon (arrow).

On presentation, he had a positive hook test, and the distal biceps tendon could not be palpated in his antecubital fossa. His neurovascular status was intact. MRI showed the complete rupture of the distal biceps tendon insertion associated with an 8.5 cm proximal retraction and mild tendinosis with partial tearing at the common extensor tendon origin (Figure 1). The patient was informed regarding his options of conservative and surgical repair versus reconstruction and the patient elected surgical repair of the distal biceps tendon. Surgery was performed two months after the initial injury.

Surgical technique

**Figure 2:**
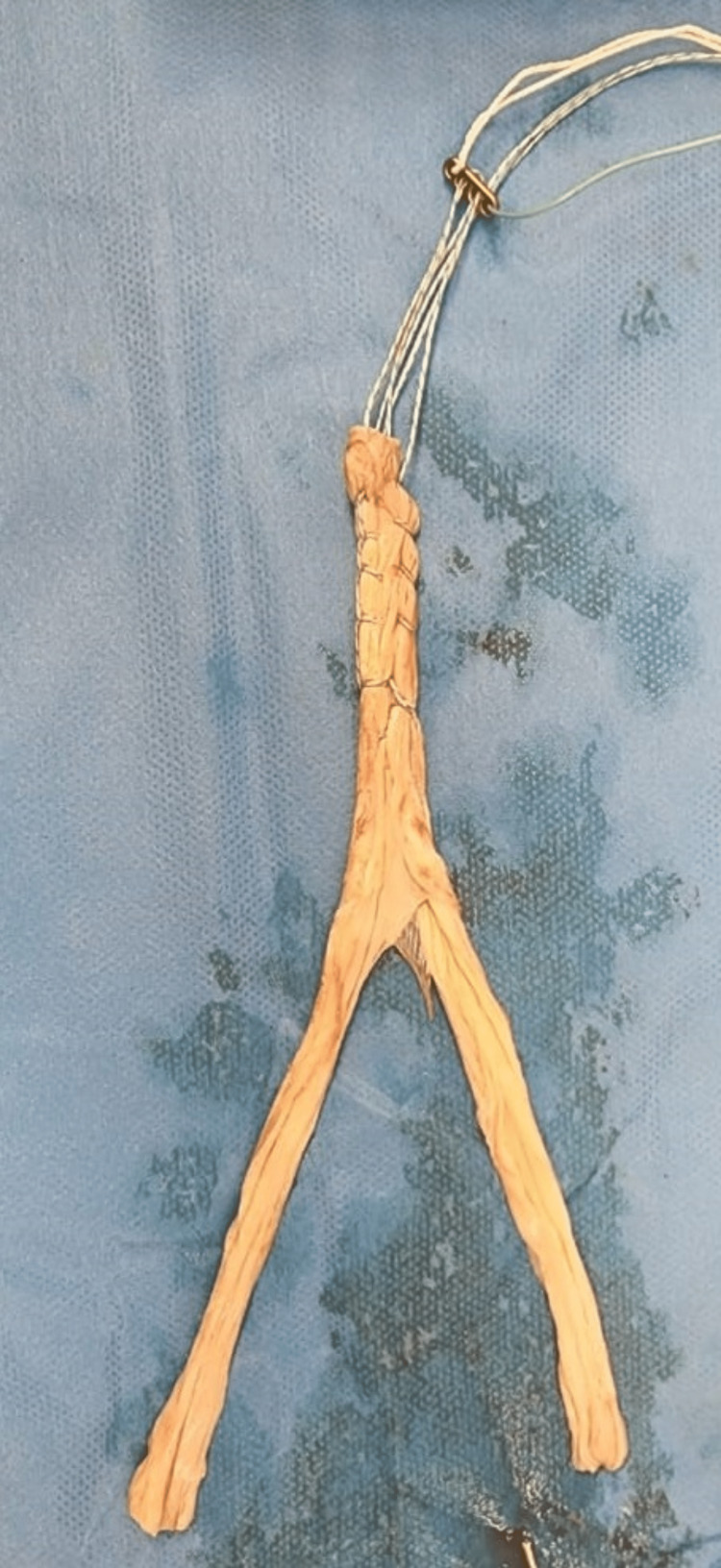
Tendon graft attached to the suture button.

**Figure 3:**
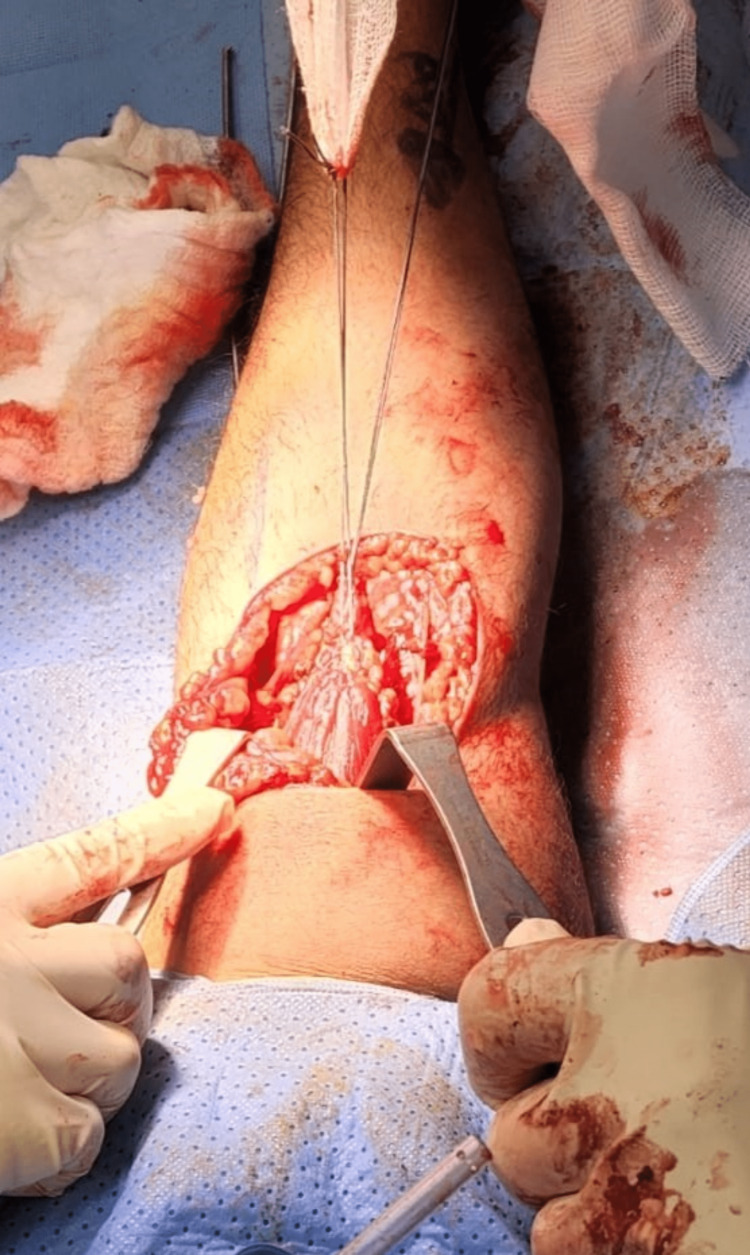
Intraoperative image showing the fixation of the graft to the ruptured biceps tendon.

**Figure 4:**
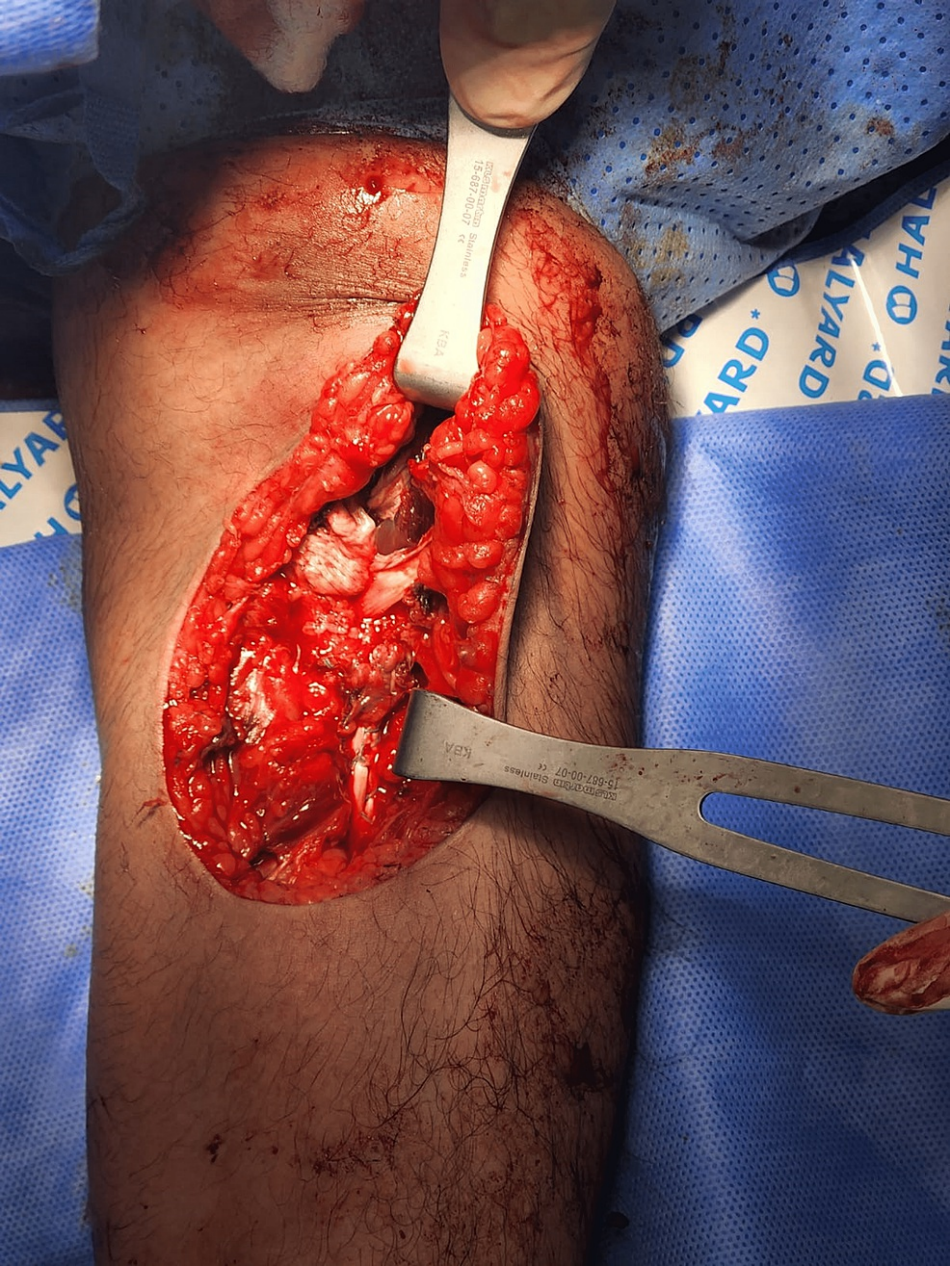
Intraoperative image demonstrating the final result of the repaired distal tendon.

**Figure 5:**
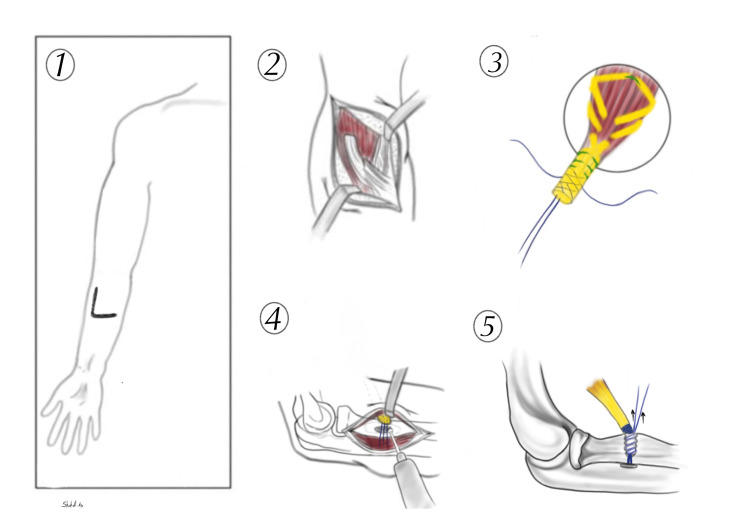
Illustration of the surgical technique. The sutures strands are pulled to dunk the tendon at the base of the tunnel (5).

The patient underwent general anesthesia in a supine position and his extended left arm was placed over a hand table. A non-sterile tourniquet was applied over the upper arm, and a horizontal incision was made distal to the antebrachial crease and then extended proximally on the medial aspect of the arm. A superficial dissection was then made to identify and protect the lateral antebrachial nerve as well as the antebrachial neurovascular structures and a branch of the musculocutaneous nerve. Finger dissection and release were done and the lacertus fibrosis was released. Subsequently, the ruptured distal biceps tendon was identified and debrided to identify healthy tissue, and the scar was released to mobilize the tendon. The radial tuberosity was then exposed with gentle dissection, and a blunt dissection was used to follow the course of the torn distal biceps to the radial tuberosity. The tendon was tagged with high-strength polyethylene suture, size two, and tension was applied to relax the muscle. A gap of more than 8.5 cm while the elbow was flexed to 60 degrees was noted. Due to the retraction of the tendon, an over-the-shelf freeze-dried tibialis posterior allograft was used. The graft was soaked in normal saline and vancomycin for 30 minutes. During this time, the tourniquet was released and radial tuberosity was prepared in full supination, drilled, and reamed unicortically to size 7 mm according to graft size. An anterior cruciate ligament fixed loop button was used and the sutures were taken out to have a free button. The allograft was whipstitched and sutures were passed through and back through the holes of the button to help pull the tendon through the tunnel. The button was then flipped and the sutures were passed through the tendon and a knot was tightened (Figure 2). A split was made on the other end of the allograft to give a Y shape. The elbow was held flexed at a 45-degree position to re-establish tension, and the split ends of the allograft were weaved through the tendon and muscle and tied together proximally (Figure 3). We checked the elbow range of motion and ensured that no excessive tension was applied during the repair (Figure 4). Fluoroscopy confirmed the proper placement of the button. Irrigation and closure of superficial layers were done, and the arm was placed in a brace. The patient tolerated the procedure without complications. The surgical technique is illustrated in Figure 5.

## Discussion

Biceps tendon ruptures are rare. Chronic use of systemic steroids, growth hormones, and tobacco has been associated with tendon rupture, as shown in our case. Wrenn et al. [4] showed that corticosteroid therapy delayed the regeneration of the tendon sheath, decreased the formation of fibrous tissue, and reduced the strength of repaired tendons by 40%. Chronic corticosteroid use leads to tendon stiffness and less energy absorption and is more likely to fail during elongation [5].

Treatment of chronic distal biceps tendon rupture can be conservative or surgical. However, there is an agreement in the literature that surgical repair of chronic ruptures is generally preferred due to the higher functional outcome, regardless of whether a single or double incision is used. It has been demonstrated that patients treated conservatively experience up to a 30% reduction in flexion power and a 40% reduction in supination power [6]. Surgical reattachment of the distal biceps tendon to the bicipital tuberosity is considered the gold standard treatment, especially in young people with high functional demands. Chronic ruptures associated with tendon retraction and scarring make the surgery more problematic and prone to complications. This leaves the surgeon with a few options such as repair in flexion, non-anatomic repair of the distal biceps to the brachialis muscle, and distal biceps tendon reconstruction.

In comparison to non-anatomic repairs, anatomic repairs are now used wherever possible. This has been well documented in the literature. Klonz et al. [7] showed reduced supination strength with anatomic repairs (91% of the contralateral side) compared with non-anatomic repairs (ranging from 42% to 56% of the contralateral side). Rantanen et al. [8]. carried out a meta-analysis of 147 cases and reported that 90% of anatomic repairs showed excellent results, although only 60% of non-anatomical repairs did. Moreover, Schmidt et al. [9] showed that when the forearm was placed between neutral and full supination with the repair anterior to the normal anatomic attachment, supination was significantly reduced.

Anatomic repairs can be challenging in chronic or delayed ruptured distal biceps, as in this case, due to tendon or muscle shortening and adhesion formation which make it more prone to complications. Radial nerve palsy and heterotopic bone formation are the common complications of surgery [10]. Our patient underwent distal biceps tendon reconstruction with an allograft. During the follow-up, the patient regained full supination, flexion, and extension without complications. The patient started gradual active full range of motion exercises and strengthening from the sixth postoperative week. At the three-month follow-up, he regained his full range of motion and strength and was able to return to his daily life activities. The patient was satisfied with the treatment from an aesthetic and functional point of view.

## Conclusions

This case emphasizes the importance of educating adult patients about the risk factors of tendon rupture, especially those who take systemic steroids for an extended period. Early surgical intervention in distal biceps tendon rupture must also be emphasized for a simple repair with a lower risk of complications. Chronic distal biceps tendon rupture with a short tendon stump can be managed using allograft reconstruction, as described here and in other papers with excellent short-term outcomes in our patient.
